# cGMP and NHR Signaling Co-regulate Expression of Insulin-Like Peptides and Developmental Activation of Infective Larvae in *Strongyloides stercoralis*

**DOI:** 10.1371/journal.ppat.1004235

**Published:** 2014-07-10

**Authors:** Jonathan D. Stoltzfus, Stephen M. Bart, James B. Lok

**Affiliations:** 1 Department of Pathobiology, University of Pennsylvania School of Veterinary Medicine, Philadelphia, Pennsylvania, United States of America; 2 Department of Biology, Hollins University, Roanoke, Virginia, United States of America; Uniformed Services University, United States of America

## Abstract

The infectious form of the parasitic nematode *Strongyloides stercoralis* is a developmentally arrested third-stage larva (L3i), which is morphologically similar to the developmentally arrested dauer larva in the free-living nematode *Caenorhabditis elegans*. We hypothesize that the molecular pathways regulating *C. elegans* dauer development also control L3i arrest and activation in *S. stercoralis*. This study aimed to determine the factors that regulate L3i activation, with a focus on G protein-coupled receptor-mediated regulation of cyclic guanosine monophosphate (cGMP) pathway signaling, including its modulation of the insulin/IGF-1-like signaling (IIS) pathway. We found that application of the membrane-permeable cGMP analog 8-bromo-cGMP potently activated development of *S. stercoralis* L3i, as measured by resumption of feeding, with 85.1±2.2% of L3i feeding in 200 µM 8-bromo-cGMP in comparison to 0.6±0.3% in the buffer diluent. Utilizing RNAseq, we examined L3i stimulated with DMEM, 8-bromo-cGMP, or the DAF-12 nuclear hormone receptor (NHR) ligand Δ7-dafachronic acid (DA)—a signaling pathway downstream of IIS in *C. elegans*. L3i stimulated with 8-bromo-cGMP up-regulated transcripts of the putative agonistic insulin-like peptide (ILP) -encoding genes *Ss-ilp-1* (20-fold) and *Ss-ilp-6* (11-fold) in comparison to controls without stimulation. Surprisingly, we found that Δ7-DA similarly modulated transcript levels of ILP-encoding genes. Using the phosphatidylinositol-4,5-bisphosphate 3-kinase inhibitor LY294002, we demonstrated that 400 nM Δ7-DA-mediated activation (93.3±1.1% L3i feeding) can be blocked using this IIS inhibitor at 100 µM (7.6±1.6% L3i feeding). To determine the tissues where promoters of ILP-encoding genes are active, we expressed promoter::*egfp* reporter constructs in transgenic *S. stercoralis* post-free-living larvae. *Ss-ilp-1* and *Ss-ilp-6* promoters are active in the hypodermis and neurons and the *Ss-ilp-7* promoter is active in the intestine and a pair of head neurons. Together, these data provide evidence that cGMP and DAF-12 NHR signaling converge on IIS to regulate *S. stercoralis* L3i activation.

## Author Summary

Human parasitic nematodes, including *Strongyloides stercoralis*, cause extensive morbidity in the developing world. The infectious form of *S. stercoralis* is a developmentally arrested third-stage larva (L3i), which resumes development into a parasitic adult upon entering a host. The molecular mechanisms controlling the developmental arrest and activation of L3i are not well understood. The free-living nematode *Caenorhabditis elegans* has a morphologically similar developmentally arrested third-stage dauer larva, which is regulated by four canonical dauer signaling pathways. Using *C. elegans* as a model, we hypothesized that cyclic guanosine monophosphate (cGMP) signaling would be important for L3i activation and would also regulate downstream insulin/IGF-1-like signaling (IIS). Indeed, we found that the membrane-permeable cGMP analog 8-bromo-cGMP stimulated L3i activation, accompanied by an increase in transcripts of putative agonistic insulin-like peptides (ILPs), which encode the ligands for IIS. Using the *C. elegans* model, we also hypothesized that DAF-12 nuclear hormone receptor (NHR) signaling would be downstream of IIS during L3i activation. Surprisingly, we found that during L3i activation, parallel cGMP and DAF-12 NHR signaling pathways co-regulate the downstream IIS pathway via modulation of ILPs. Together, these data help to further elucidate the pathways governing *S. stercoralis* L3i development.

## Introduction

Parasitic nematodes infect approximately one in four persons globally, with the vast burden of disease concentrated in tropical and developing regions [1]. The parasitic nematode *Strongyloides stercoralis* infects an estimated 30–100 million people worldwide [2]; in corticosteroid-treated or human T-cell lymphotropic virus 1 (HTLV-1) infected persons, infection with *S. stercoralis* can result in hyperinfection and potentially fatal disseminated strongyloidiasis [3]. Like many soil-transmitted helminths, the infectious form of *S. stercoralis* is a developmentally arrested third-stage larva (L3i), which is non-feeding, long-lived, and stress-resistant [4]. *S. stercoralis* L3i exhibit thermotaxis and chemotaxis in response to a range of host-like cues [5], including host body temperature [6], carbon dioxide [7], sodium chloride [8], and urocanic acid [9]. Upon entering a suitable host, L3i quickly activate and resume feeding and development [4]. However, the molecular mechanisms by which *S. stercoralis* L3i sense and transduce host cues and subsequently initiate resumption of development are poorly understood.

**Figure 1:**
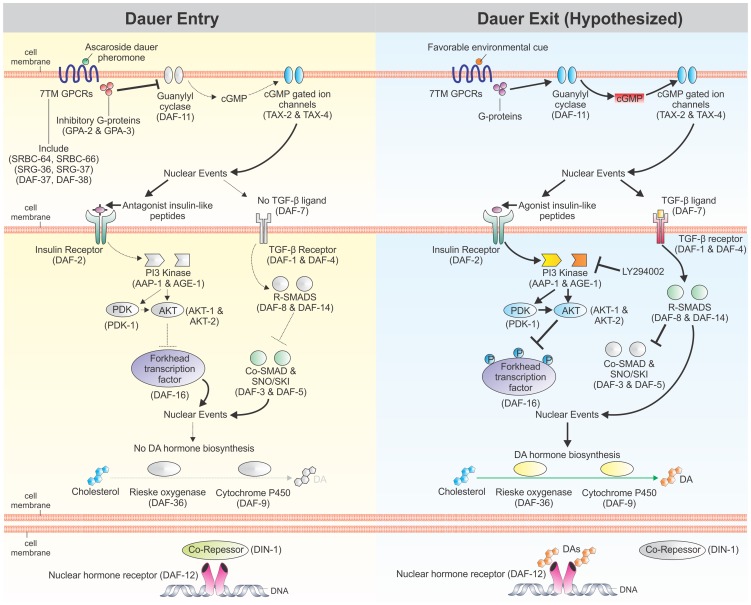
Regulation of *C. elegans* dauer development by cellular signaling pathways. Dauer entry in *C. elegans* is regulated by four signaling pathways: a cyclic guanosine monophosphate (cGMP) signaling pathway, an insulin/IGF-1-like signaling (IIS) pathway, a dauer transforming growth factor β (TGFβ) signaling pathway, and a DAF-12 nuclear hormone receptor (NHR) regulated by dafachronic acid (DA) steroid ligands. Unfavorable conditions stimulate dauer entry (left panel) by down-regulating cGMP production, increasing expression of antagonistic insulin-like peptides that down-regulate IIS, decreasing expression of the dauer TGFβ ligand, and inhibiting production of DAs. When dauer larvae encounter favorable conditions, these pathways are hypothesized to act in reverse. Dotted lines signify down-regulated signaling through the pathway, while black lines signify up-regulated signaling through the pathway. Colored proteins are active; grayed out proteins are inactive. Grayed out ligands are absent. Adapted from [12], [32], [43], [91].

The free-living nematode *Caenorhabditis elegans* has a facultative developmentally arrested third-stage larva, known as the dauer larva, which forms during stressful conditions including high temperature, low food abundance, and high dauer pheromone levels; when conditions improve, *C. elegans* exits dauer and resumes reproductive development [10], [11]. The molecular pathways regulating dauer entry have been well studied and include: a cyclic guanosine monophosphate (cGMP) signaling pathway, an insulin/IGF-1-like signaling (IIS) pathway, a dauer transforming growth factor β (TGFβ) signaling pathway, and a DAF-12 nuclear hormone receptor (NHR) regulated by dafachronic acid (DA) steroid ligands (Figure 1) [12], [13]. We have demonstrated that components of these four pathways are present in *S. stercoralis* [14], that elements of the IIS pathway control L3i arrest and activation [15], [16], and that Δ7-DA is a potent activator of L3i [17]. However, a role for cGMP signaling in regulating *S. stercoralis* L3i development has not been previously examined, nor have the epistatic relationships of these pathways been explored.

In *C. elegans*, cGMP is a second messenger for many chemosensory seven-transmembrane G protein-coupled receptors (7TM GPCRs), which act as sensors for a wide variety of environmental stimuli and regulate the developmental switch controlling dauer versus reproductive development. Indeed, the *C. elegans* genome encodes over 1,300 chemosensory 7TM GPCRs [18], [19], which signal through the 21 G protein α (G_α_) subunits, two G protein β (G_β_) subunits, and two G protein γ (G_γ_) subunits encoded in the genome [20]. One of the primary functions of these G proteins is to regulate guanylyl cyclases, including *Ce*-DAF-11, which in turn produce cGMP [21]. Downstream effects of cGMP signaling are mediated, in part, by heteromeric cyclic-nucleotide gated ion channels, including the one formed by *Ce*-TAX-4 and *Ce*-TAX-2, which regulates chemosensation, thermosensation, and dauer development [22]–[24].

Perhaps one of the best studied examples of chemosensory 7TM GPCRs regulating development through cGMP signaling is the sensing of dauer pheromone, which is a measure of population density in *C. elegans* [25]. Dauer pheromone, a complex mixture of ascarosides [26]–[29], is continuously secreted by *C. elegans*, and high concentrations potently induce dauer formation [11], [30]. Several chemosensory 7TM GPCRs sense specific or combinations of dauer-inducing ascarosides; these include *Ce*-SRBC-64 and *Ce*-SRBC-66 [31], *Ce*-SRG-36 and *Ce*-SRG-37 [32], and *Ce*-DAF-37 and *Ce*-DAF-38 [33]. At least two of these chemosensory 7TM GPCRs signal through the G_α_ subunits *Ce*-GPA-2 and *Ce*-GPA-3 [31]. When dauer pheromone levels are high, *Ce*-GPA-2 and *Ce*-GPA-3 inhibit the transmembrane guanylyl cyclase encoded by *Ce-daf-11*, thereby decreasing concentrations of the second messenger cGMP [31]. Constitutively-activated forms of *Ce*-GPA-2 or *Ce*-GPA-3, as well as inactivating mutations in *Ce-daf-11*, result in dauer constitutive (Daf-c) phenotypes, while mutations that inactivate *Ce*-GPA-2 or *Ce*-GPA-3 result in decreased dauer entry under dauer-inducing conditions [34]–[37].

Exogenous application of the membrane-permeable cGMP analog 8-bromo-cGMP rescues the Daf-c phenotype of *Ce-daf-11* mutants, but does not rescue the Daf-c phenotypes of *Ce-tax-4*, IIS pathway, or dauer TGFβ pathway mutants [36]. Furthermore, addition of 8-bromo-cGMP increases abundance of *Ce-ins-7* and *Ce-daf-28* transcripts [38], which both encode agonistic insulin-like peptides (ILPs) in the IIS pathway [39], [40]. Signaling mediated by cGMP also regulates the dauer TGFβ pathway, since *Ce-daf-11* acts cell autonomously to regulate *Ce-daf-7* expression in ASI chemosensory neurons [41], [42]. These data suggest that cGMP signaling acts upstream of both IIS and TGFβ signaling in regulating *C. elegans* dauer development.

Genetic epistatic analysis of the four pathways regulating dauer development has placed *Ce*-DAF-12 NHR signaling downstream of cGMP, IIS, and dauer TGFβ signaling [12], [34], [43]–[46]. *Ce*-DAF-12 is an NHR regulated by DA steroid ligands, the presence of which promotes reproductive growth and development [47], [48]. Exogenous application of Δ7-DA can rescue the Daf-c phenotype of both IIS and dauer TGFβ pathway mutants [47]. Additionally, both IIS and dauer TGFβ pathways regulate *Ce*-DAF-9, which encodes a cytochrome P450 that catalyses the rate-limiting final step in DA biosynthesis [49], [50]. Together, these data suggest that *Ce*-DAF-12 NHR signaling is the downstream effector for *C. elegans* dauer development.

While the pathways regulating *C. elegans* dauer development are well-studied, the mechanisms regulating L3i developmental arrest and activation in parasitic nematodes are comparatively not well understood. In particular, the roles of cGMP signaling and the epistatic relationships of canonical dauer pathways in regulating *S. stercoralis* L3i arrest and activation have not been examined. Previous work has demonstrated that increased cGMP signaling activates L3i in *Ancylostoma* and *Nippostrongylus* hookworms (clade 9B) [51]–[54], which are closely related to *C. elegans* (clade 9A) [54]; however, this has not been demonstrated in *S. stercoralis* (clade 10B) [54], where parasitism is thought to have arisen independently of the hookworms [55]. Additionally, next-generation deep-sequencing of the transcriptome (RNAseq) of *S. stercoralis* revealed increased transcript levels of many cGMP signaling pathway components in L3i [14]. Together, these data led us to hypothesize that chemosensory 7TM GPCRs sense host cues and that cGMP pathway signaling is important for transducing host-like cues in *S. stercoralis* L3i as well as triggering resumption of development once inside the host.

Although we and others have investigated the role of canonical dauer pathways in regulating L3i arrest and activation [14], [56], we are not aware of any studies examining the epistatic relationship of these pathways in parasitic nematodes. While studies examining the role of dauer TGFβ signaling in parasitic nematodes have largely concluded that this pathway regulates L3i development differently from *Ce*-DAF-7 [57]–[62], studies examining IIS and DAF-12 NHR signaling suggest these pathways function similarly in regulating both L3i and dauer development [15], [17], [63]–[65]. Therefore, we also hypothesized that the epistatic ordering of cGMP, IIS, and DAF-12 NHR signaling pathways regulating *C. elegans* dauer development would be retained in *S. stercoralis* during L3i activation.

In this study, we sought to determine the roles of and the relationships between canonical dauer pathways in the regulation of *S. stercoralis* L3i activation. We found that during L3i activation, parallel cGMP and DAF-12 NHR signaling pathways co-regulate the downstream IIS pathway via modulation of ILPs. Understanding the mechanisms of L3i activation may lead to new or improved therapies for parasitic nematode infections.

## Methods

### Ethics statement

The *S. stercoralis* PV001 and UPD strains were maintained in prednisone-treated beagles in accordance with protocols 702342, 801905, and 802593 approved by the University of Pennsylvania Institutional Animal Care and Use Committee (IACUC). All IACUC protocols, as well as routine husbandry care of the animals, were carried out in strict accordance with the *Guide for the Care and Use of Laboratory Animals of the National Institutes of Health*.

### *S. stercoralis* L3i *in vitro* activation

The *S. stercoralis* PV001 and UPD strains were maintained and cultured as previously described [16], [66], [67].

*In vitro* activation of *S. stercoralis* L3i was performed as previously described [16], [68] with the following adaptations. All conditions were supplemented with antibiotics (100 U/ml penicillin, 10 µg/ml streptomycin, and 12.5 µg/ml tetracycline). M9 Buffer was used as the medium for both the experimental conditions and the negative control [69], except where indicated.

For the titration of 8-bromo-cGMP, L3i were isolated from six-day-old charcoal coprocultures (incubated at 25°C) by the Baermann technique at 27°C. L3i were subsequently washed twice in deionized water and incubated in M9 buffer supplemented with antibiotics for three hours at room temperature before distribution amongst the different conditions. The positive control was composed of Dulbecco's Modification of Eagle's Medium (DMEM) (supplemented with L-glutamine, 4.5 g/L glucose, and sodium pyruvate) (Corning, www.cellgro.com), 10% naive canine serum, and 12.5 mM L-glutathione reduced (CAS 70-18-8) (Sigma-Aldrich, www.sigmaaldrich.com). The negative control was M9 buffer. The experimental conditions, using 8-bromo-cGMP (CAS 51116-01-9) (Sigma) at 800 µM, 200 µM, 100 µM, and 50 µM, were carried out in M9 buffer. Each condition consisted of three wells in a 96-well plate, with approximately 100 L3i in 100 µl total volume in each well. L3i were incubated at 37°C in 5% CO_2_ in air for 21 hours; 2.5 µl of fluorescein isothiocyanate (FITC; CAS 3326-32-7) (Sigma) dissolved in N,N-dimethylformamide (CAS 68-12-2) (Sigma) at 20 mg/ml and incubated for ≥ one month was then added to each well, and the cultures were incubated an additional three hours at 37°C and 5% CO_2_ in air (24 hours total). L3i for each condition were pooled and washed five times in 14 ml of M9 buffer, with centrifugation at 75× g for five minutes at room temperature. L3i were then mounted on glass slides with grease-edged cover-slips, immobilized by a 20-second heat-shock at 60°C or with 10 mM levamisole (CAS 16595-80-5) (Sigma), and viewed by fluorescence microscopy. Only L3i with FITC in the pharynx were scored as “positive” for feeding. Three biological replicates were performed, and the mean percentage of L3i feeding in each condition with the standard deviation was plotted in Prism version 5.03 (GraphPad Software, Inc., http://www.graphpad.com/).

For L3i activation kinetics, conditions included both 200 µM 8-bromo-cGMP in M9 buffer and host-like cues, consisting of DMEM, 10% canine serum, and 3.75 mM L-glutathione reduced. L3i were isolated as previously described. L3i in all conditions were incubated at 37°C and 5% CO_2_ in air for a total of 24 hours, with chemical cues added at appropriate intervals such that L3i were exposed to activating compounds for 4, 6, 12, 18, or 24 hours total. L3i were incubated with FITC and scored for feeding as previously described. Three biological replicates were performed, and the mean percentage of L3i feeding in each condition with the standard deviation was plotted in Prism.

For titration of Δ7-DA, conditions included Δ7-DA at 800 nM, 400 nM, 200 nM, 100 nM, and 50 nM in M9 buffer as well as an M9 buffer negative control. Additionally, 100 µM LY294002, a phosphatidylinositol-4,5-bisphosphate 3-kinase (PI3 kinase) inhibitor previously demonstrated to inhibit L3i activation [16], in 1.3% dimethyl sulfoxide (DMSO) was mixed with 400 nM Δ7-DA in M9 buffer; the negative control was 1.3% DMSO in M9 buffer. L3i were isolated as previously described. L3i in all conditions were incubated at 37°C and 5% CO_2_ in air for a total of 24 hours. L3i were incubated with FITC and scored for feeding as previously described. Four biological replicates were performed, and the mean percentage of L3i feeding in each condition with the standard deviation was plotted in Prism.

For RNAseq analysis, PV001 strain L3i were isolated from seven-day-old charcoal coprocultures (incubated at 25°C) by the Baermann technique at 27°C. L3i were subsequently washed twice in deionized water and incubated in M9 buffer supplemented with antibiotics for three hours at room temperature. An aliquot of these L3i (no stimulation control) was pelleted and frozen in 100 µl TRIzol reagent (Life Technologies, www.lifetechnologies.com). L3i were then distributed amongst the following conditions, each supplemented with antibiotics: M9 buffer, DMEM (supplemented with L-glutamine, 4.5 g/L glucose, and sodium pyruvate), 200 µM 8-bromo-cGMP in M9 buffer, and 400 nM Δ7-DA in M9 buffer. We found that removal of the reduced glutathione and naive canine serum components from the DMEM-based biochemical mixture had no effect on L3i activation; thus, fresh DMEM without these additives was used. L3i were incubated at 37°C and 5% CO_2_ in air for a total of 24 hours using 500 L3i in 100 µl of liquid in each well of a 96-well round-bottom plate with 24 wells per condition. One well from each condition was used to assess the percentage of L3i ingesting the FITC dye, as previously described. L3i from the remaining 23 wells were pooled and the pellet frozen at −80°C in 100 µl TRIzol reagent. This protocol was repeated for a total of four biological replicates. The mean percentage of L3i feeding in each condition with the standard deviation was plotted in Prism.

### *S. stercoralis* RNAseq

*S. stercoralis* L3i were activated for RNAseq analysis as described, with a total of four biological replicates for each of the five conditions. The L3i pellet in TRIzol was thawed and ground using a pestle, and total RNA was extracted per the manufacturer's protocol. RNA concentrations were determined using a NanoDrop 2000 spectrophotometer (Thermo Scientific, www.nanodrop.com). Total RNA was additionally quantified using the Bioanalyzer 2100 (Agilent Technologies, Inc., http://www.agilent.com), and only samples with an RNA integrity number (RIN) greater than 8.0 were used.

Libraries were constructed using the TruSeq RNA Sample Preparation Kit v2 (Illumina, Inc., http://www.illumina.com) according to the manufacturer's protocol. For each library, 500 ng of total RNA, diluted to 10 ng/µl in de-ionized water, was used as starting material. Polyadenylated RNA enrichment was performed first using olido-dT beads and eluted polyadenylated RNA fragmented at 94°C for eight minutes to approximately 200±65 (standard deviation) bp. Subsequently, first and second strand cDNA was synthesized; unique adapters for each replicate were then ligated. dsDNA fragments with ligated adapters were enriched using 14 cycles of PCR. Libraries were assessed for fragment size distribution using the Bioanalyzer 2100.

The concentration of the dsDNA adapter-ligated libraries was then determined by quantitative PCR (qPCR) with the Kapa SYBR Fast qPCR Kit for Library Quantification (Kapa Biosystems, Inc., http://www.kapabiosystems.com) using the manufacturer's protocol. Three dilutions, at 1∶4,000, 1∶8,000, and 1∶16,000, were used to calculate the concentration of each of the libraries using a calibration curve of Kapa standards. Each library was then diluted to 10 nM and pooled in equal volume quantities.

Pools were sequenced on the Illumina HiSeq 2000 with 100 bp paired-end reads, with image analysis and base calling performed with HiSeq Control Software. Raw flow-cell data was processed and demultiplexed using CASAVA (Illumina) for each of the 21 samples. Raw RNAseq reads are available in the ArrayExpress database (www.ebi.ac.uk/arrayexpress) under accession number E-MTAB-2192.

### Alignment of *S. stercoralis* RNAseq reads to genomic contigs

Raw reads from each L3i activation sample were independently aligned to *S. stercoralis* genomic contigs (6 December 2011 draft; ftp://ftp.sanger.ac.uk/pub/pathogens/HGI/) using TopHat2 version 2.0.9 (http://tophat.cbcb.umd.edu/) [70], which utilized the Bowtie2 aligner version 2.1.0 (http://bowtie-bio.sourceforge.net/bowtie2/index.shtml) [71] and SAMtools version 0.1.19 (http://samtools.sourceforge.net/). Default parameters were used, but with the following options: mate inner distance of 25; mate standard deviation of 50; minimum anchor length of 6; minimum intron length of 30; maximum intron length of 20,000; micro exon search; minimum segment intron of 30; and maximum segment intron of 20,000. Aligned reads from each developmental stage were inspected using the Integrated Genome Viewer (IGV) version 2.3.20 (http://www.broadinstitute.org/igv/).

Additional *S. stercoralis* developmental stages used for RNA isolation, dsDNA library construction and sequencing (ArrayExpress accession number E-MTAB-1164; http://www.ebi.ac.uk/arrayexpress/experiments/E-MTAB-1164), and read alignment to the *S. stercoralis* draft genome (6 December 2011 version) have been described previously [14]. *De novo* assembly of RNAseq reads from *S. stercoralis* developmental stages has also been described previously [14]. These seven developmental stages include: free-living females (FL Female), post-free-living first-stage larvae (PFL L1), infectious third-stage larvae (L3i), *in vivo* activated third-stage larvae (L3+), parasitic females (P Female), post-parasitic first-stage larvae (PP L1), and post-parasitic third-stage larvae (PP L3).

### Differential analysis of *S. stercoralis* transcripts

Transcript abundances of manually annotated *S. stercoralis* genes were calculated using Cufflinks version 2.0.2 (http://cufflinks.cbcb.umd.edu/) as fragments per kilobase of coding exon per million fragments mapped (FPKM), with paired-end reads counted as single sampling events [72]. FPKM values for coding sequences (CDS) (Data S1, S2, S3), along with ±95% confidence intervals, were calculated for each gene using Cuffdiff version 2.0.2 (Data S4). FPKMs and 95% confidence intervals were plotted in Prism. Significant differences in FPKM values between developmental stages and p-values were determined using Cuffdiff version 2.0.2, a program with the Cufflinks package [73], [74]; p-values less than 0.05 were considered statistically significant.

### Identification of *S. stercoralis* GPCRs and G proteins

BLAST searches of the *S. stercoralis* (ftp://ftp.sanger.ac.uk/pub/pathogens/HGI/) genomic contigs, as well as *S. stercoralis de novo* assembled transcripts (ArrayExpress accession number E-MTAB-1184; http://www.ebi.ac.uk/arrayexpress/experiments/E-MTAB-1184), using *C. elegans* protein sequences (Data S5) were performed using Geneious version 6.0 (www.geneious.com) set to the least restrictive parameters. A total of 76 *C. elegans* chemosensory 7TM GPCRs, four from each of the 19 families (Data S5), were used to BLAST search the *S. stercoralis* genomic contigs, resulting in a total of 227 hits. The 21 *C. elegans* G_α_ proteins, the two G_β_ proteins, and the two G_γ_ proteins (Data S5), were used to search both the *S. stercoralis* genomic contigs and *de novo* assembled transcripts.

BLAST hits were manually annotated using aligned reads from all seven developmental stages by a combination of IGV and Geneious. Putative *S. stercoralis* homologs were identified through reverse BLAST searches using NCBI's pBLAST (http://blast.ncbi.nlm.nih.gov/Blast.cgi) [75] against *C. elegans* sequences. Putative *S. stercoralis* homologs of chemosensory 7TM GPCRs were also checked for transmembrane domains using TMHMM Server version 2.0 (http://www.cbs.dtu.dk/services/TMHMM/) and assigned to a *C. elegans* superfamily based on reverse BLAST search results. Manually annotated *S. stercoralis* transcripts were used to determine predicted protein sequences.

Phylogenetic analysis of *C. briggsae*, *C. elegans*, and *S. stercoralis* G_α_ proteins was performed by alignment of protein sequences with Clustal W and a BLOSUM matrix using Geneious (Data S6). A neighbor-joining phylogenetic tree, with 1000 iterations of bootstrapping, was then constructed using Geneious. *S. stercoralis* G_α_ protein-encoding genes were named by relationship to *C. briggsae* and *C. elegans* proteins (Figure S1).

### Plasmid construction and transformation of *S. stercoralis*

Plasmids for microinjection of *S. stercoralis* were constructed by Gateway cloning technology (Life Technologies). In general, DNA sequences between the start codons of genes of interest and the stop codons of the genes immediately upstream were used as promoters in *Ss-ilp* reporter transgene constructs. The *Ss-ilp-1* promoter region, containing 2,327 bp 5′ of the start site, was PCR amplified from *S. stercoralis* genomic DNA, using the primers Ssilp1-1F and Ssilp1-1R, and recombined into pDONR P4-P1R, forming pPV483. The *Ss-ilp-6* promoter region, containing 2,566 bp 5′ of the start site, was PCR amplified from *S. stercoralis* genomic DNA, using the primers Ssilp6-1F and Ssilp6-1R, and recombined into pDONR P4-P1R, forming pPV484. The *Ss-ilp-7* promoter region, containing 1,270 bp 5′ of the start site, was PCR amplified from *S. stercoralis* genomic DNA, using the primers Ssilp7-1F and Ssilp7-1R, and recombined into pDONR P4-P1R, forming pPV485. The 870 bp coding sequence and stop codon for enhanced green fluorescent protein (*egfp*) was PCR amplified from pJA257 (Addgene, www.addgene.org), using the primers EGFPGW-1F and EGFPGW-1R, and recombined into pDONR 221, forming pPV477. The *Ss-era-1* terminator, consisting of 598 bp 3′ of *Ss-era-1* [76], was PCR amplified from pAJ08 (Addgene), using the primers Ss-era-1-1FattB2r and Ss-era-1-1RattB3, and recombined into pDONR P2R-P3, forming pPV475. Primer sequences are listed in Data S7. *S. stercoralis* genomic DNA was prepared from mixed-stage worms using the Qiagen DNeasy Blood and Tissue kit (www.qiagen.com). All pDONR plasmid inserts were confirmed by complete sequencing.

*S. stercoralis* ILP promoter::*egfp* reporter plasmids were constructed by LR recombination reactions using Gateway. The *Ss-ilp-1* promoter::*egfp*::*Ss-era-1* terminator plasmid was constructed by recombining the plasmids pPV483, pPV477, and pPV475 into pDEST R4-R3, forming pPV487. The *Ss-ilp-6* promoter::*egfp*::*Ss-era-1* terminator plasmid was constructed by recombining the plasmids pPV484, pPV477, and pPV475 into pDEST R4-R3, forming pPV488. The *Ss-ilp-7* promoter::*egfp*::*Ss-era-1* terminator plasmid was constructed by recombining the plasmids pPV485, pPV477, and pPV475 into pDEST R4-R3, forming pPV489. All pDEST plasmid inserts were confirmed by complete sequencing.

*S. stercoralis* was transformed by gonadal micro-injection of adult free-living females as previously described [66]. A mix of 50 ng/µl of either pPV487, pPV488, or pPV489 and 20 ng/µl of pAJ08 (Addgene) as a co-injection marker was micro-injected into the distal gonad of gravid females. Injected females were paired with an equal number of males and incubated on an NGM agar plate, with *E. coli* OP50 as a food source, at 22°C. The F1 post-free-living progeny were screened for fluorescence both 48 and 72 hours after microinjection. Larvae were screened for expression of fluorescent reporter transgenes using an Olympus SZX12 stereomicroscope with coaxial epifluorescence (www.olympus.com). Each transgenic larva was subsequently mounted on a 2% agarose pad (Lonza, www.lonza.com), anesthetized with 10 mM levamisole (Sigma), and examined in detail using an Olympus BX60 compound microscope equipped with Nomarski Differential Interference Contrast (DIC) optics and epifluorescence. Specimens were imaged using a Spot RT Color digital camera and Spot Advanced v5.1 image analysis software (Diagnostic Instruments, Inc., www.spotimaging.com). Captured images were processed using GIMP version 2.6 (www.gimp.org) and Microsoft PowerPoint 2007 (www.microsoft.com).

## Results

### *S. stercoralis* chemosensory 7TM GPCRs

We previously discovered that in *S. stercoralis*, transcripts of genes encoding putative cGMP signaling proteins are up-regulated in L3i, and so we speculated that cGMP signaling may be important in L3i for relaying host cues and controlling resumption of development upon entering a permissive host [14]. Since chemosensory 7TM GPCRs are known to regulate cGMP signaling and to be crucial for *C. elegans*' response to environmental cues [18], [19], [77], including the sensing of ascarosides [31]–[33], we hypothesized that homologs in *S. stercoralis* might play a role in sensing environmental and host cues, especially in L3i. Thus, we surveyed homologs of chemosensory 7TM GPCRs in *S. stercoralis* to determine whether the transcripts are developmentally regulated in a manner consistent with a role in sensing host cues.

Using reciprocal BLAST searches, with four disparate members from each of the 19 *C. elegans* chemosensory 7TM GPCR families used to conduct the initial search [19], we identified a total of 85 genes in the *S. stercoralis* genome that encode putative chemosensory 7TM GPCRs (Table 1). These 85 putative *S. stercoralis* chemosensory 7TM GPCRs almost certainly compose an incomplete list of the total number of *S. stercoralis* chemosensory 7TM GPCRs; however, this list likely includes the majority of these receptors encoded in the genome, given the open parameters of the BLAST searches. The chemosensory 7TM GPCRs from *S. stercoralis* were assigned by sequence homology to the SRA, SRG, SRSX, and STR superfamilies of 7TM GPCRs (Table 1). Although we included four members from each of the *C. elegans* SRW, SRZ, and SRBC superfamilies in our BLAST searches, we did not find homologs from any of these superfamilies in *S. stercoralis*. This search also identified other conserved classes of 7TM receptors (data not shown). We determined which of the seven *C. elegans* superfamilies each of the *S. stercoralis* chemosensory 7TM GPCRs were homologous to using BLAST scores (Table 1). However, we were unable to assign *S. stercoralis* homologs to specific *C. elegans* families due to large predicted protein sequence differences.

**Table 1:** Transcript abundance of *S. stercoralis* homologs of *C. elegans* chemosensory 7TM GPCRs.

*C. elegans* superfamily	Transcripts in L3i only	Transcripts in L3+ only	Transcripts in L3i & L3+	Transcripts in other stages	Transcripts not detected	Total number
***Ss*** **-SRA**	1	1	3	1	5	11
***Ss*** **-SRG**	6*	2	9	7	29	53*
***Ss*** **-SRSX**	-	-	-	1	1	2
***Ss*** **-STR**	5	2	2	1	9	19

*Includes one gene with a premature “stop” codon.

Utilizing RNAseq data from seven different *S. stercoralis* developmental stages [14], we determined the transcript abundance patterns for each of the 85 putative *S. stercoralis* chemosensory 7TM GPCR homologs (Table 1). Surprisingly, nearly all of the transcript abundance profiles fit into one of only four patterns: transcripts detected only in L3i, only in *in vivo* activated L3+, in both L3i and L3+, or in none of the developmental stages examined. Furthermore, the normalized transcript abundance, calculated as FPKM, for nearly all of these transcripts was lower than for many other genes we have examined in *S. stercoralis*.

### *S. stercoralis* heterotrimeric G proteins

In *C. elegans*, as well as other metazoans, chemosensory 7TM GPCRs signal through heterotrimeric G proteins to intracellular effectors [20], [78]. G proteins are composed of three separately transcribed peptides: the G_α_ subunit that interacts directly with the 7TM GPCR and confers functional specificity, the G_β_ subunit, and the G_γ_ subunit [21]. The *C. elegans* genome contains 21 G_α_ subunit-, two G_β_ subunit-, and two G_γ_ subunit-encoding genes; the promoters for the majority of the G_α_ subunit-encoding genes are active in chemosensory amphidial neurons [20]. Two of the *C. elegans* G_α_ subunit-encoding genes, *Ce-gpa-2* and *Ce-gpa-3*, play a role in larval commitment to dauer development [31], [37]. Our lab has previously identified orthologs of these two genes in *S. stercoralis*, named *Ss-gpa-2* and *Ss-gpa-3*, the transcripts of which are at a maximum in L3i [14], [79]. Additionally, the promoter for *Ss-gpa-3* is active in the amphidial neurons of transgenic *S. stercoralis* post-free-living larvae, suggesting that it plays a role in relaying chemosensory cues [80]. Thus, we sought to identify all the G proteins in *S. stercoralis* and examine their transcript abundance patterns to determine whether the transcripts of other G proteins in the parasite are also at a maximum in L3i.

Using reciprocal BLAST searches, we identified a total of 14 G_α_ subunit-, two G_β_ subunit-, and two G_γ_ subunit-encoding genes in *S. stercoralis* (Table 2). By comparing the putative protein sequences for the *S. stercoralis* G_α_ subunits with *C. elegans* and *C. briggsae* sequences in a phylogenetic analysis, we were able to determine the G_α_ gene class [78] for each of the *S. stercoralis* G_α_ subunits as well as their orthologous relationships with their *Caenorhabditis* spp. counterparts (Table 2, Figure S1). Notably absent from the *S. stercoralis* genomic contigs, as well as the *de novo* assembled transcripts, were orthologs for *gpa-1*, *-8*, *-9*, *-11*, *-14*, *-15*, and *-16*.

**Table 2:** Identification of *S. stercoralis* heterotrimeric G protein orthologs.

G protein	*C. elegans*	*S. stercoralis*	*S. stercoralis* transcript abundance profile
**G_α_ subunits**			
**G_ns_**	*Ce*-GPA-1	np	
	*Ce*-GPA-2	*Ss*-GPA-2	peak in L3i
	*Ce*-GPA-3	*Ss*-GPA-3	peak in L3i
	*Ce*-GPA-7	*Ss*-GPA-7	peak in L3i
	*Ce*-GPA-8	np	
	*Ce*-GPA-9	np	
	*Ce*-GPA-10	*Ss*-GPA-10	peak in L3i
	*Ce*-GPA-15	np	
	*Ce*-ODR-3	*Ss*-ODR-3	peak in L3i
**G_nsd_**	*Ce*-GPA-11	np	
	*Ce*-GPA-13	*Ss*-GPA-13	peak in L3i
	*Ce*-GPA-14	np	
	*Ce*-GPA-17	*Ss*-GPA-17	present in all stages examined
**G_i/o_**	*Ce*-GOA-1	*Ss*-GOA-1	present in all stages examined
	*Ce*-GPA-4	*Ss*-GPA-4	absent in L3i
	*Ce*-GPA-16	np	
**G_q_**	*Ce*-EGL-30	*Ss*-EGL-30	present in all stages examined
**G_s_**	*Ce*-GSA-1	*Ss*-GSA-1	present in all stages examined
**G_12_**	*Ce*-GPA-5	*Ss*-GPA-5	peak in L3i
	*Ce*-GPA-6	*Ss*-GPA-6	only detected in PFL L1, L3i, and L3+
	*Ce*-GPA-12	*Ss*-GPA-12	peak in L3i
**G_β_ subunits**	*Ce*-GPB-1	*Ss*-GPB-1	present in all stages examined
	*Ce*-GPB-2	*Ss*-GPB-2	present in all stages examined
**G_γ_ subunits**	*Ce*-GPC-1	*Ss*-GPC-1	present in all stages examined
	*Ce*-GPC-2	*Ss*-GPC-2	nadir in P female

Note: G_ns_, nematode-specific G_α_ subunit; G_nsd_, divergent nematode-specific G_α_ subunit; np, not present in the *S. stercoralis* genomic contigs or *de novo* assembled transcripts.

We then examined the transcript abundance patterns for each of the G protein-encoding genes, using RNAseq data from seven *S. stercoralis* developmental stages [14]. We found that for many of the nematode-specific G_α_ subunit orthologs [78], transcript abundance reached a peak in L3i (Table 2, Figure S2). In contrast, transcripts for *Ss-gpa-4* were detected in all developmental stages examined, except L3i. Transcripts from the highly conserved G_β_ subunit and G_γ_ subunit genes were found in all developmental stages examined (Table 2, Figure S3).

### 8-bromo-cGMP activates *S. stercoralis* L3i

In previous studies, we observed an increase in transcripts encoding guanylyl cyclases in *S. stercoralis* L3i [14], suggesting that when L3i encounter a permissive host, one of the downstream effects of chemosensory 7TM GPCR signaling through heterotrimeric G proteins is an increase in the second-messenger cGMP. A similar pathway—and accompanying increase in cGMP—has been described in *C. elegans* in response to odorants [81]. Additionally, other research groups have used the membrane-permeable analog of cGMP, 8-bromo-cGMP, to test whether increases in cGMP can activate L3i in place of host-like cues in *Ancylostoma caninum* [52], *Ancylostoma ceylanicum* [51], and *Nippostrongylus brasiliensis* [53]. In these three hookworm species (clade 9B) [54], which are closely related to *C. elegans* (clade 9A), 8-bromo-cGMP activates L3i at 5 mM for *A. caninum* and *A. ceylanicum* and at 500 µM for *N. brasiliensis*.

Parasitism in *S. stercoralis* (clade 10B) is thought to have arisen independently of the hookworm species [55]. For this reason, we asked whether increases in cGMP can also activate L3i in place of host-like cues in this parasite. To this end, we applied 8-bromo-cGMP to *S. stercoralis* L3i and assessed activation in an *in vitro* feeding assay [16], [68]. We incubated *S. stercoralis* L3i in a range of 8-bromo-cGMP concentrations in M9 buffer, M9 buffer as a negative control, and a mixture of biochemical host-like cues, consisting of DMEM supplemented with 10% canine serum and 12.5 mM reduced glutathione, as a positive control, for a total of 24 hours at 37°C and 5% CO_2_ in air; we then assessed resumption of feeding, a hallmark of activation, by ingestion of a FITC fluorescent dye.

**Figure 2:**
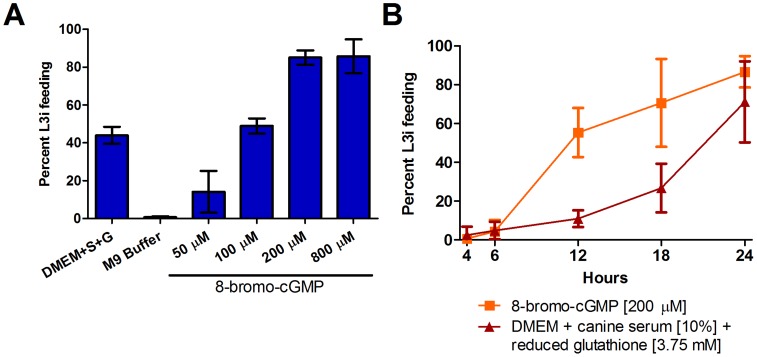
*S. stercoralis* L3i are activated by 8-bromo-cGMP. The membrane-permeable cGMP analog, 8-bromo-cGMP, induced resumption of feeding, a hallmark of activation, in *S. stercoralis* L3i. Feeding was assessed by ingestion of a FITC dye after incubation at 37°C and 5% CO_2_ in air for 24 hours for all conditions. (A) At 200 µM, 8-bromo-cGMP dissolved in M9 buffer results in potent resumption of feeding in L3i, with 85.1% (±2.2, SD) of larvae feeding after 24 hours. In comparison, host-like cues consisting of DMEM, 10% canine serum (S), and 12.5 mM reduced glutathione (G), resulted in 43.9% (±2.6, SD) of L3i feeding, while the M9 buffer negative control resulted in 0.6% (±0.3, SD) of L3i feeding, after 24 hours. (B) Kinetics of activation were determined for both 200 µM 8-bromo-cGMP and host-like cues, consisting of DMEM, 10% canine serum, and 3.75 mM reduced glutathione, after incubation for 4, 6, 12, 18, or 24 hours. All conditions were incubated for a total of 24 hours at 37°C and 5% CO_2_ in air. Error bars represent ±1 standard deviation (SD).

*S. stercoralis* L3i were activated by 8-bromo-cGMP, with a concentration of 200 µM resulting in 85.1% (±2.2%, SD) of L3i feeding compared to 0.6% (±0.3%, SD) for the M9 buffer negative control (Figure 2A). At much higher concentrations of 8-bromo-cGMP (i.e. 2 mM or greater), we observed L3i that were radially constricted in alternating segments along the longitudinal axis and that had a compromised cuticle, as evidenced by permeability to the FITC dye (data not shown).

To compare the kinetics of activation of *S. stercoralis* L3i by host-like cues to L3i activated by 8-bromo-cGMP, we examined the frequency of feeding over a 24-hour time course. We determined the percentage of L3i feeding after incubation in 200 µM 8-bromo-cGMP or a mixture of DMEM, 10% canine serum, and 3.75 mM reduced glutathione, for 4, 6, 12, 18, and 24 hours at 37°C and 5% CO_2_ in air. We found that 8-bromo-cGMP activated L3i more rapidly than the mixture of biochemical host-like cues (Figure 2B).

### RNAseq analysis of *S. stercoralis* L3i activation

**Figure 3:**
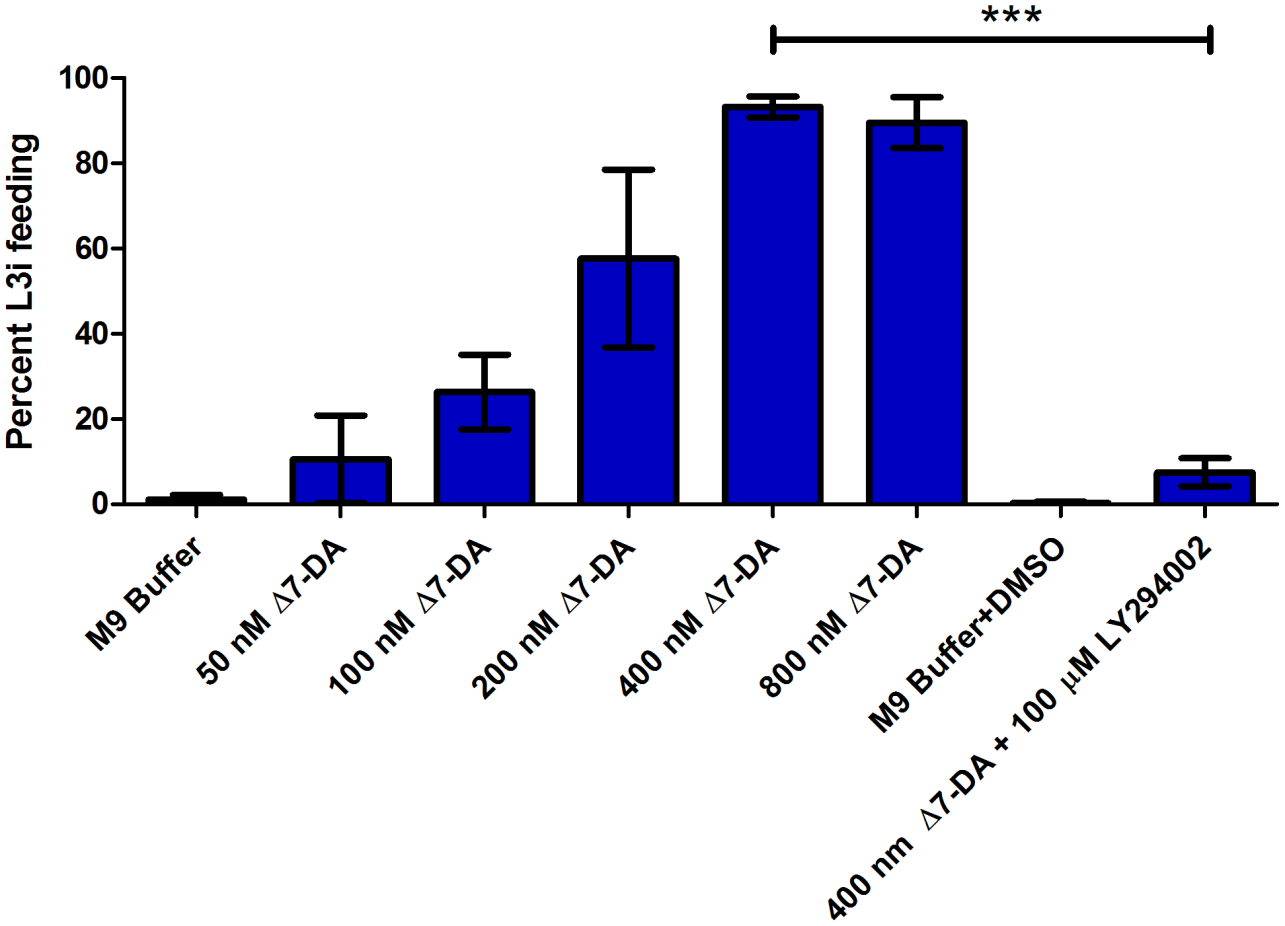
*S. stercoralis* L3i activation with Δ7-DA and inhibition with the PI3 kinase inhibitor LY294002. The putative DAF-12 nuclear hormone receptor ligand Δ7-dafachronic acid (DA) induced resumption of feeding, a hallmark of activation, in *S. stercoralis* L3i in a dose-dependent manner. Feeding was assessed by ingestion of a FITC dye after incubation at 37°C and 5% CO_2_ in air for 24 hours for all conditions. Conditions included Δ7-DA at 800 nM, 400 nM, 200 nM, 100 nM, and 50 nM dissolved in M9 buffer. At 400 nM Δ7-DA, 93.3% (±1.1%, SD) of L3i resumed feeding in comparison to 1.2% (±0.4%) in the M9 buffer control. Additionally, the phosphatidylinositol-4,5-bisphosphate 3-kinase (PI3 kinase) inhibitor LY294002 was added to Δ7-DA to determine whether inhibition of PI3 kinases would block activation by Δ7-DA. At 100 µM, LY294002 dissolved in DMSO inhibited L3i activation in 400 nM Δ7-DA with 7.6% (±1.6%, SD) of L3i feeding in this condition; 0.28% (±0.2%) of L3i had resumed feeding in the M9 buffer with DMSO negative control. Error bars represent ±1 standard deviation (SD); *** p<0.01.

To investigate changes in the abundance of transcripts during L3i activation, we utilized RNAseq to examine the differences in parasites exposed to five different conditions. To control for the influence of host-like temperature on L3i activation, we used L3i exposed to neither thermal nor biochemical host-like cues (no stimulation control) and L3i exposed to thermal cues only (M9 buffer control). These two control conditions were compared to L3i stimulated with both thermal and chemical host-like cues. Titration of Δ7-DA in M9 buffer demonstrated that frequency of L3i feeding was maximal at 400 nM (93.3±1.1%, SD; Figure 3); at concentrations of Δ7-DA 2 µM or greater, we observed a large percentage of L3i that were immobilized, curled, or dead (as evidenced by cuticle permeability to the FITC dye). Thus, we used the following conditions, in addition to thermal cues, to stimulate L3i: DMEM alone, 200 µM 8-bromo-cGMP, and 400 nM Δ7-DA. We hypothesized that these three activating conditions (DMEM, 8-bromo-cGMP, and Δ7-DA) would exhibit different activation profiles, since they target different parts of the L3i activation pathway.

**Figure 4:**
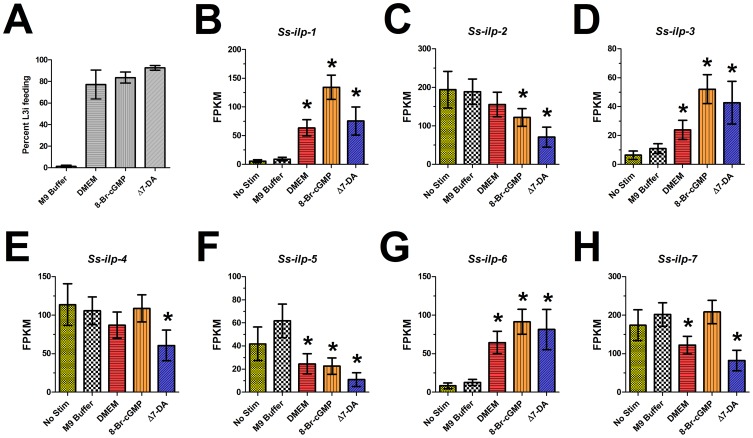
*S. stercoralis* L3i activation with 8-bromo-cGMP, Δ7-DA, or DMEM modulates ILP ligand transcript levels. Transcript levels of the insulin-like peptide (ILP) ligand-encoding genes *Ss-ilp-1* through *Ss-ilp-7* were quantified using RNAseq. Conditions included L3i that had no stimulation (only exposed to room temperature conditions in M9 buffer) as well as L3i incubated at 37°C and 5% CO_2_ in air for 24 hours in either M9 buffer, DMEM, 200 µM 8-bromo-cGMP in M9 buffer, or 400 nM Δ7-dafachronic acid (DA) in M9 buffer. (A) L3i feeding, a hallmark of activation, was assessed by ingestion of a FITC dye for worms incubated at 37°C and 5% CO_2_ in air for 24 hours. Error bars represent ±1 standard deviation. (B–H) Transcript abundance patterns for *Ss-ilp-1* through *-7* were determined by RNAseq for each condition. Transcript abundances were calculated as fragments per kilobase of coding exon per million fragments mapped (FPKM). Error bars represent ±95% confidence intervals. All statistically significant differences, with respect to the no stimulation condition, are marked with an asterisk.

Using four biological replicates, we assessed the percentage of L3i feeding, as measured by ingestion of FITC dye, in each of the conditions incubated for 24 hours (Figure 4A). RNA was extracted from each of the five conditions for each of the four biological replicates, and RNAseq libraries were constructed. We then determined the normalized transcript abundance (FPKM) for each gene of interest.

### Transcripts encoding ILPs are regulated during activation of L3i by 8-bromo-cGMP

Studies in *C. elegans* have revealed that cGMP pathway signaling lies upstream of IIS and regulates transcript levels of *Ce-daf-28* and *Ce-ins-7*, both proposed to be agonists of the *Ce*-DAF-2 insulin-like receptor [38], [39]. Previously, we identified seven ILPs in *S. stercoralis* and noted that the transcripts for several of these are developmentally regulated [14]. In *A. caninum*, 8-bromo-cGMP activation has been correlated with the transcriptional profile observed with serum stimulation; however, no canonical dauer pathway component transcripts were examined in this study [82]. To determine whether cGMP signaling also regulates transcripts encoding ILPs during activation of *S. stercoralis* L3i, we utilized RNAseq to examine changes in transcript levels for *Ss-ilp-1* through *-7* following L3i activation with 8-bromo-cGMP (Figure 4).

Several of the *S. stercoralis* ILP-encoding transcripts were significantly regulated during L3i activation (Figure 4). Based on their predicted protein sequences and transcript abundance patterns in different developmental stages, we previously hypothesized that both *Ss-ilp-1* and *Ss-ilp-6* encode agonistic ILPs while *Ss-ilp-7* encodes an antagonistic ILP for the DAF-2 insulin-like receptor [14]. Compared to the no stimulation control, *Ss-ilp-1* transcripts were increased over 20-fold in L3i stimulated with 8-bromo-cGMP (p<0.01). Additionally, *Ss-ilp-3* and *Ss-ilp-6* transcripts increased significantly (8-fold and 11-fold, respectively) in L3i stimulated with 8-bromo-cGMP compared to the no stimulation control (p<0.01). By contrast, levels of *Ss-ilp-7* transcripts following 8-bromo-cGMP activation were unchanged relative to the no stimulation or M9 buffer controls. Interestingly, DMEM-mediated activation of L3i resulted in modulation of ILP-encoding transcripts similar to activation with 8-bromo-cGMP.

Studies in *C. elegans* have shown that cGMP pathway signaling also regulates the dauer TGFβ pathway, including transcript levels of the single dauer TGFβ ligand-encoding gene *Ce-daf-7* [41]. We previously described seven *daf-7*-like genes in *S. stercoralis*, named *Ss-tgh-1* through *-7*, and noted that *Ss-tgh-1*, *-2*, and *-3* transcripts were only detected in L3i [14], [61]. We examined the changes in transcript abundance for *Ss-tgh-1* through *-7* upon L3i stimulation with 8-bromo-cGMP (Figure S4). We observed significant decreases in transcript abundance for *Ss-tgh-1*, *-2*, and *-3* in 8-bromo-cGMP-treated worms in comparison to the M9 buffer control (p<0.01). Interestingly, *Ss-tgh-6* was up-regulated ≥14-fold in 8-bromo-cGMP-treated worms in comparison to either the no stimulation or M9 buffer controls (p<0.01).

### Transcripts encoding ILPs are regulated during activation of L3i by Δ7-DA

Epistatic analysis in *C. elegans* has placed DAF-12 NHR signaling downstream of the cGMP, IIS, and dauer TGFβ pathways with respect to regulation of dauer development [12], [34], [83]. Operating under the assumption that this epistatic relationship also exists during *S. stercoralis* L3i activation, we hypothesized that activation of L3i with Δ7-DA would not modulate ILP transcripts, since NHR signaling is downstream of IIS in *C. elegans*. To our surprise, Δ7-DA regulated ILP-encoding transcripts in a manner similar to 8-bromo-cGMP, which is upstream of IIS in *C. elegans* (Figure 4). We found that *Ss-ilp-1*, *Ss-ilp-3*, and *Ss-ilp-6* transcripts were significantly increased (14-fold, 7-fold, and 10-fold, respectively) in L3i stimulated with Δ7-DA in comparison to the no stimulation control (p<0.01), similar to their regulation by 8-bromo-cGMP. These results suggest that NHR signaling may be upstream of IIS during *S. stercoralis* L3i activation.

We hypothesized that regulation of ILP-encoding transcripts by Δ7-DA was either a general phenomenon that would be observed with any activating condition or that IIS signaling was downstream of NHR signaling in *S. stercoralis* during L3i activation. The only two stimuli other than Δ7-DA that, to our knowledge, result in L3i feeding (DMEM and 8-bromo-cGMP) are both predicted to signal upstream of both IIS and NHR signaling; thus, we were unable to directly test whether regulation of ILP-encoding transcripts by Δ7-DA was a general phenomenon observed with any activating condition. However, we hypothesized that if NHR signaling was indeed upstream of IIS, we would be able to block Δ7-DA-mediated activation of L3i by inhibiting IIS. In previous work, we demonstrated that the PI3 kinase inhibitor LY294002 blocks L3i activation at 100 µM [16]. Using our *in vitro* L3i activation assay, we found that 100 µM LY294002 in the presence of 400 nM Δ7-DA resulted in 7.6% (±1.6%, SD) of L3i feeding in comparison to 93.3% (±1.1%, SD) in 400 nM Δ7-DA alone. Thus, this PI3 kinase inhibitor almost completely blocked the effect of Δ7-DA-mediated activation in comparison to 0.3% (±0.2%, SD) feeding in the M9 buffer+DMSO negative control (Figure 3).

### *S. stercoralis* ILP promoters are active in the nervous system and other tissues

Activation of *S. stercoralis* L3i by administration of 8-bromo-cGMP or Δ7-DA results in regulation of ILP-encoding transcripts similar to that observed in third-stage larvae three days after infection of a permissive host [14]. Furthermore, this regulation of ILP-encoding transcripts is accompanied by a phenotype, namely resumption of feeding. *C. elegans* ILPs regulate IIS not only by changes in transcript abundance, but also by localization of their expression in specific tissues. For this reason, given the evidence that IIS appears to play a critical role in mediating L3i arrest and activation, we identified the tissues in which the promoters of several *S. stercoralis* ILP-encoding genes are active. In *C. elegans*, the promoters of genes encoding ILPs are often active in the nervous system, intestine, and/or gonad [39], [84]. These tissues are important regulators of dauer development, longevity, and responsiveness to environmental stresses [39], [84], [85]. Previously, we reported that the transcript abundances of three *S. stercoralis* ILPs, *Ss-ilp-1*, *-6*, and *-7*, are differentially regulated during post-free-living development [14]. To determine whether these three *S. stercoralis* ILPs are expressed in similar tissues as *C. elegans* ILPs, we made promoter::*egfp* reporter constructs for *Ss-ilp-1*, *-6*, and *-7* and expressed these in transgenic *S. stercoralis* post-free-living larvae.

**Figure 5:**
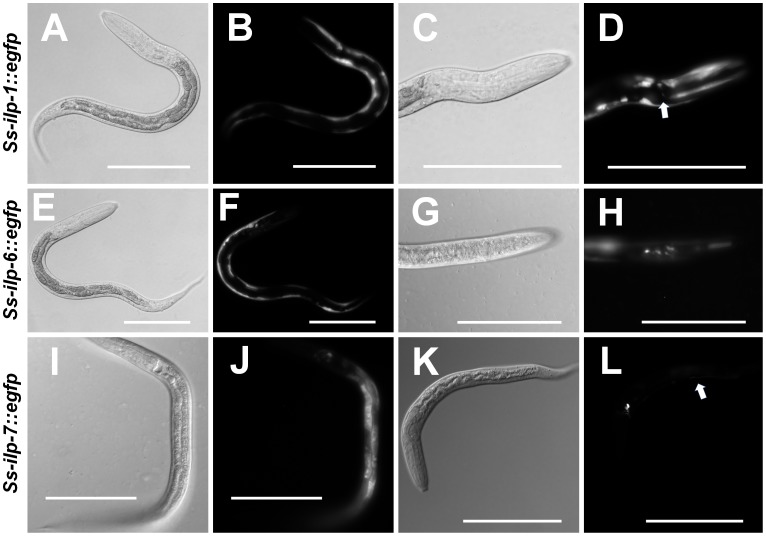
*S. stercoralis* ILP promoters are active in the nervous system and other tissues. Transgenic *S. stercoralis* post-free-living larvae expressing enhanced green fluorescent protein (EGFP) under the control of three insulin-like peptide (ILP) promoters were assessed for tissue-specific expression. (A–D) Transgenic larvae carrying the *Ss-ilp-1* promoter::*egfp* reporter construct; (A,C) DIC images and (B,D) fluorescent images. The *Ss-ilp-1* promoter is active in the hypodermis/body wall and a single pair of head neurons (D, arrow). (E–H) Transgenic larvae carrying the *Ss-ilp-6* promoter::*egfp* reporter construct; (E,G) DIC images and (F,H) fluorescent images. The *Ss-ilp-6* promoter is active in the hypodermis/body wall and several head neurons. (I–L) Transgenic larvae carrying the *Ss-ilp-7* promoter::*egfp* reporter construct; (I,K) DIC images and (J,L) fluorescent images. The *Ss-ilp-7* promoter is active in the intestine and a single pair of head neurons, with a single process that extends dorsally almost to the anterior portion of the intestine (L, arrow), most consistent with the SIAV neurons in *C. elegans*.

We observed EGFP under control of the *Ss-ilp-1* promoter in the hypodermis/body wall as well as a single pair of head neurons (Table 3, Figure 5A–D). The promoter activity for *Ss-ilp-6* was similar to *Ss-ilp-1*, with EGFP observed in the hypodermis/body wall and head neurons; however, *Ss-ilp-6* promoter activity was observed in several pairs of head neurons and tail neuron(s), while *Ss-ilp-1* promoter activity was limited to a single pair of head neurons (Table 3, Figure 5E–H). EGFP under control of the *Ss-ilp-7* promoter was localized to the intestine as well as a single pair of head neurons with a single process that extended dorsally almost to the anterior portion of the intestine (Table 3, Figure 5I–L). The location and shape of this pair of neurons is most consistent with SIAV in *C. elegans*.

**Table 3:** Location of EGFP expression in transgenic *S. stercoralis* post-free-living larvae under the control of ILP promoters.

Promoter	Intestine	Hypodermis/body wall	Head neuron(s)	Tail neuron(s)	Pharynx	Other cell body	Total number
***Ss-ilp-1***	0 (0%)	40 (100%)	20 (50%)	1 (3%)	0 (0%)	2 (5%)	n = 40
***Ss-ilp-6***	0 (0%)	39 (100%)	20 (51%)	6 (15%)	1 (3%)	0 (0%)	n = 39
***Ss-ilp-7***	21 (100%)	1 (5%)	18 (81%)	1 (5%)	0 (0%)	1 (5%)	n = 21

## Discussion

In this study, we sought to both describe the upstream components that regulate the second messenger cGMP in *S. stercoralis*, including chemosensory 7TM GPCRs and heterotrimeric G proteins, and determine whether cGMP pathway signaling regulates *S. stercoralis* L3i activation. Additionally, we sought to elucidate the epistatic relationships between cGMP signaling, IIS, and DAF-12 NHR signaling pathways during L3i activation. We hypothesized that the cGMP-regulated chemosensory pathway may be one of the first to transduce host cues when *S. stercoralis* L3i encounter a permissive host. This hypothesis was based on our previous observation that the transcripts of multiple cGMP pathway components are increased in *S. stercoralis* L3i, suggesting that this pathway may be “poised” to transduce host cues [14], and studies demonstrating that exogenous application of 8-bromo-cGMP activates L3i of hookworm species [51]–[53]. We therefore sought to describe the components of a chemosensory 7TM GPCR signaling pathway in *S. stercoralis* and determine whether cGMP signaling regulates L3i activation as well as IIS and other signaling pathways.

Using RNAseq data from seven *S. stercoralis* developmental stages and draft *S. stercoralis* genomic contigs [14], we identified and characterized the developmental transcript profiles for 85 chemosensory 7TM GPCRs predicted in the *S. stercoralis* genome. The majority of chemosensory 7TM GPCR-encoding transcripts were found in L3i and/or L3+ (Table 1) at abundances that were low compared to other *S. stercoralis* transcripts. These data strongly suggest that these receptors act in a few chemosensory cells to sense host cues. Other chemosensory 7TM GPCR-encoding transcripts in *S. stercoralis*, which were not observed in this study, may be present in life stages such as the free-living male or autoinfective L3 (L3a), which have not yet been interrogated by RNAseq. In these stages, the encoded chemosensory 7TM GPCRs might transduce chemical signals important in mate finding or migration within the host, respectively.

The paucity of chemosensory 7TM GPCR-encoding genes in *S. stercoralis* in comparison to *C. elegans* is not entirely surprising given the evolutionary distance separating *S. stercoralis* (clade 10B) and *C. elegans* (clade 9A) [54] and the large differences in the number of chemosensory 7TM GPCRs among even closely related nematode species [19]. The differences in the number of chemosensory 7TM GPCR genes within the well-studied *Caenorhabditis* genus is illustrated by the fact that there are approximately 40% more chemosensory 7TM GPCR-encoding genes in the *C. elegans* genome (1,646 genes) than in the *C. briggsae* genome (1,151 genes) [19]. Thus, the chemosensory 7TM GPCR family of receptors appears to have a great deal of evolutionary plasticity in terms of absolute number and ligand specificity. Additionally, *S. stercoralis*, like many parasitic nematodes, predominately resides inside a nutrient-rich host or in bacteria-rich feces and thus does not need to continually navigate and adapt to a complex external environment with limited resources. The need for chemosensory 7TM GPCRs in *S. stercoralis* is mainly limited to L3i sensing a host, larval migration within the host, and free-living male and female mate attraction during heterogonic development. These differences in lifestyle between *S. stercoralis* and *C. elegans* may partially account for the smaller number of 7TM GPCR-encoding genes in *S. stercoralis*. Whether this reduction in the number of 7TM GPCR-encoding genes is a feature of all nematode parasites or only *S. stercoralis* is currently unknown.

We used a similar strategy to identify G proteins in *S. stercoralis*; however, the sequence divergence between *S. stercoralis* and *C. elegans* of the proteins these genes encode is far less than for the chemosensory 7TM GPCRs. By phylogenetic analysis, we were able to identify the *C. elegans* ortholog for each of the *S. stercoralis* G_α_-, G_β_-, and G_y_-encoding genes (Table 2, Figures S1, S2, & S3). Interestingly, *S. stercoralis* appears to have only 14 G_α_-encoding genes in comparison to the 21 in *C. elegans*. This observation is congruous with the smaller number of chemosensory 7TM GPCRs in *S. stercoralis*, since fewer receptors would need fewer signal transduction molecules. Many of the nematode-specific G_α_-encoding genes have transcripts that are at their peak in L3i (Table 2, Figure S2). Along with our previous observation that the *Ss-gpa-3* promoter is active in amphidial neurons [80], these data are consistent with a role for *S. stercoralis* G_α_ subunits in relaying environmental and host chemosensory cues in L3i.

Using a previously established *S. stercoralis* L3i feeding assay [16], [68], we demonstrated that exogenous application of the membrane-permeable cGMP analog 8-bromo-cGMP stimulates L3i activation (Figure 2A) with a higher potency than that observed in experiments with other parasitic nematodes [51]–[53]. Furthermore, 8-bromo-cGMP activated L3i more quickly than a mixture of host-like biochemical cues (Figure 2B). These data suggest that increases in endogenous cGMP levels accompany *S. stercoralis* L3i activation upon encountering a permissive host. Since previous work has only demonstrated 8-bromo-cGMP activation in hookworm species (clade 9B) [51]–[53], which are closely related to *C. elegans* (clade 9A) [54], these findings from the distantly related *S. stercoralis* (clade 10B), where parasitism is thought to have arisen independently from the hookworm species [55], suggest a more broadly conserved mechanism of L3i activation in parasitic nematodes.

Although cGMP signaling appears to be involved in L3i activation of both hookworms and *S. stercoralis*, this is a departure from the role of this pathway in regulating *C. elegans* dauer arrest when dauer pheromone is present. In *C. elegans*, elevated levels of ascarosides, which accompany high population density, bind chemosensory 7TM GPCRs that activate the inhibitory G proteins *Ce*-GPA-2 and *Ce*-GPA-3 that repress the guanylyl cyclase *Ce*-DAF-11, ultimately decreasing cGMP levels and promoting dauer entry [31]. While ascaroside pheromones have been detected in many nematode species [86]–[88], it is difficult to envision a role for these compounds in regulating L3i development for the parasitic nematodes of many warm-blooded animals, particularly in species where all post-parasitic larvae invariably developmentally arrest in the infectious form [89]. In *S. stercoralis*, where post-parasitic larvae can facultatively develop to a single generation of free-living males and females, the post-free-living larvae invariably develop to L3i regardless of population density. In these cases, an L3i-promoting ascaroside seems unlikely. However, a dauer-like pheromone that regulates L3i formation has recently been described in the parasitic nematode *Parastrongyloides trichosuri*, which can undergo multiple rounds of free-living replication outside its animal host [90]. Thus, cGMP pathway signaling may have several roles in free-living and parasitic nematodes, including modulation of L3i and dauer development by transduction of favorable (e.g., host or food) as well as unfavorable (e.g., dauer pheromone) environmental cues.

The second aim of this study was to determine the epistatic relationships of cGMP signaling, IIS, and DAF-12 NHR signaling in regulating *S. stercoralis* L3i activation. We hypothesized that, as in *C. elegans*, cGMP signaling would be upstream of IIS and that DAF-12 NHR signaling would be downstream of IIS [12], [34]. In *C. elegans*, cGMP signaling regulates both the IIS pathway as well as the dauer TGFβ pathway, including modulation of their cognate peptide ligands [38], [39], [41]. Using RNAseq, we demonstrated that activation of *S. stercoralis* L3i by 8-bromo-cGMP was accompanied by a dramatic increase in *Ss-ilp-1* and *Ss-ilp-6* transcripts (Figure 4B and G). In previous work, we described *Ss*-ILP-1 as a putative IIS agonist, due to protein sequence similarities with *C. elegans* agonistic ILPs and a decrease in *Ss-ilp-1* transcript levels during parasitic development [14]; thus, our data suggest that *Ss-ilp-1* transcripts increase immediately following L3i activation, but then decrease again during development to the parasitic female, consistent with a role as an agonistic ILP. We similarly described *Ss*-ILP-6 as a putative IIS agonist due to protein sequence similarities with *C. elegans* agonistic ILPs and an increase in *Ss-ilp-6* transcripts in third-stage larvae that were activated inside a permissive host for three days [14]; thus, a similar regulation of *Ss-ilp-6* transcripts by administered 8-bromo-cGMP reinforces our assertion that stimulation with this compound mimics early *in vivo* L3i activation and that *Ss-ilp-6* encodes an IIS agonist important in L3i activation.

We also observed modulation of dauer TGFβ ligand transcripts during 8-bromo-cGMP-stimulated *S. stercoralis* L3i activation. Although developmental regulation of dauer TGFβ pathway homologs and an increase in the number of dauer TGFβ ligands from one to seven in *S. stercoralis* suggests a different role for this pathway than in *C. elegans*, the dauer TGFβ pathway does appear to be important in L3i, since three of the TGFβ ligands have transcripts only detected in this developmental stage [14]. Our observation that *Ss-tgh-1*, *-2*, and *-3* transcripts are all decreased following L3i activation (Figure S4B-D) is consistent with these previous findings, which infer a role for TGFβ signaling in parasitic nematodes that is opposite to its apparent role in *C. elegans* dauer regulation [57]–[61]. Together, our RNAseq results suggest that, as in *C. elegans*, cGMP pathway signaling is upstream of both IIS and dauer TGFβ signaling in *S. stercoralis*.

In *C. elegans*, DAF-12 NHR signaling is downstream of IIS in regulating dauer entry, as evidenced by genetic epistatic analysis and rescue of the daf-c phenotype in *daf-2(e1368)* worms by Δ7-DA [34], [44], [47]. Therefore, we hypothesized that Δ7-DA-mediated L3i activation would not involve modulating ILP transcripts or IIS. Surprisingly, we found that the profiles of ILP transcript abundance were almost identical in 8-bromo-cGMP-mediated and Δ7-DA-mediated L3i activation (Figure 4). We reasoned that either modulation of ILP transcripts levels, and thus IIS, is a non-specific feature of *S. stercoralis* L3i activation or that our assumption about pathway ordering was incorrect. To test this, we utilized LY294002, a potent inhibitor of PI3 kinases such as *Ss*-AGE-1, which blocks L3i feeding when activating biochemical host-like cues are present [16]. We found that LY294002 almost completely abolishes Δ7-DA-mediated L3i activation (Figure 3). While we cannot entirely account for off-target effects of LY294002, which inhibits all PI3 kinases, only three classes of PI3 kinases are present in nematodes and the class I PI3 kinase *Ce*-AGE-1 is exclusively associated with dauer development. *Ss*-AGE-1 is the ortholog of *Ce*-AGE-1 and the sole class I PI3 kinase in *S. stercoralis* [16]. Therefore, it is almost certainly the mediator of LY294002's effect. Together, the data from RNAseq and chemical inhibitor studies strongly suggest that DAF-12 NHR signaling acts upstream of IIS during L3i activation.

Since our data strongly suggest that regulation of IIS by ILPs is crucial for L3i activation, we identified the tissues in which ILP promoters are active during post-free-living development. Using previously established techniques [15], [76], [80], we expressed *S. stercoralis* ILP promoter::*egfp* reporter constructs in the post-free-living generation. We found that *Ss-ilp-1* and *Ss-ilp-6* promoters are active in head neurons as well as the hypodermis/body wall (Figure 5A–H); in previous work, we hypothesized that both of these ILPs act as IIS agonists [14]. Additionally, we found that the *Ss-ilp-7* promoter is active in both a single pair of head neurons as well as the intestine (Figure 5I–L). We previously hypothesized that *Ss-ilp-7* acts as an IIS antagonist [14]. The promoter activity of *S. stercoralis* ILPs in hypodermal, neuronal, and intestinal tissues is consistent with anatomical locations of *C. elegans* ILP promoter activity [84].

Together, our data suggest a model of *S. stercoralis* L3i activation in which parallel cGMP and DAF-12 NHR signaling co-regulate the downstream IIS pathway via modulation of ILPs. This model differs in several significant respects from current models of canonical dauer pathway regulation in *C. elegans*. First, *C. elegans* decreases cGMP pathway signaling in response to dauer pheromone, resulting in dauer arrest [31], [36]. In contrast, we hypothesize that in *S. stercoralis* L3i, host compounds bind chemosensory 7TM GPCRs, which activate G proteins that in turn activate guanylyl cyclases that increase cGMP levels; this ultimately triggers L3i to activate and resume development, in part through increased IIS via increases in agonistic ILPs [14], [16]. Second, epistatic analysis in *C. elegans* has demonstrated that dauer development is regulated by upstream cGMP signaling that regulates IIS, which in turn regulates downstream DAF-12 NHR signaling [12]. However, the data in this study suggest that both cGMP and DAF-12 NHR signaling lie upstream of IIS in regulating L3i activation, further emphasizing the importance of IIS in *S. stercoralis* L3i development.

## Supporting Information

Figure S1 **Phylogenetic analysis of** ***S. stercoralis*** **and** ***Caenorhabditis*** **spp. Gα proteins.** A protein alignment, generated with Clustal W, of *S. stercoralis* (Ss), *C. briggsae* (Cb), and *C. elegans* (Ce) heterotrimeric G protein α subunit (Gα) homologs was used to construct a neighbor-joining tree with 100 iterations of boot-strapping. Orthologs for several *C. briggsae* and *C. elegans* Gα-encoding genes (*gpa-1*, *-8*, *-9*, *-11*, *-14*, *-15*, and *-16*) were not identified in the *S. stercoralis* draft genome or *de novo* assembled transcripts. The scale bar represents substitutions per position.
(TIF)

Figure S2 **Developmental regulation of transcripts encoding** ***S. stercoralis*** **Gα subunits.** (A–N) Transcript abundance patterns in *S. stercoralis* developmental stages were determined by RNAseq for genes encoding orthologs of heterotrimeric G protein α subunits (Gα). Transcript abundances were quantified in seven developmental stages: free-living females (FL Female), post-free-living first-stage larvae (PFL L1), infectious third-stage larvae (L3i), *in vivo* activated third-stage larvae (L3+), parasitic females (P Female), post-parasitic first-stage larvae (PP L1), and post-parasitic third-stage larvae (PP L3). Transcript abundances were calculated as fragments per kilobase of coding exon per million fragments mapped (FPKM) and log transformed. Error bars represent ±95% confidence intervals. The y-axes were scaled from 0 to 3.0 to aid comparison between genes.
(TIF)

Figure S3 **Developmental regulation of transcripts encoding** ***S. stercoralis*** **G_β_ and G_γ_ subunits.** (A–D) Transcript abundance patterns in *S. stercoralis* developmental stages were determined by RNAseq for genes encoding homologs of heterotrimeric G protein β (G_β_) subunits (A,B) and γ (G_γ_) subunits (C,D). Transcript abundances were quantified in seven developmental stages: free-living females (FL Female), post-free-living first-stage larvae (PFL L1), infectious third-stage larvae (L3i), *in vivo* activated third-stage larvae (L3+), parasitic females (P Female), post-parasitic first-stage larvae (PP L1), and post-parasitic third-stage larvae (PP L3). Transcript abundances were calculated as fragments per kilobase of coding exon per million fragments mapped (FPKM) and log transformed. Error bars represent ±95% confidence intervals. The y-axes were scaled from 0 to 3.0 to aid comparison between genes.
(TIF)

Figure S4 ***S. stercoralis*** **L3i activation with 8-bromo-cGMP, Δ7-DA, or DMEM modulates TGFβ ligand transcript levels.** Transcript levels of the DAF-7-like transforming growth factor β (TGFβ) ligand-encoding genes *Ss-tgh-1* through *Ss-tgh-7* were quantified using RNAseq. Conditions included L3i that had no stimulation (only exposed to room temperature conditions in M9 buffer) as well as L3i incubated at 37°C and 5% CO_2_ in air for 24 hours in either M9 buffer, DMEM, 200 µM 8-bromo-cGMP in M9 buffer, or 400 nM Δ7-dafachronic acid (DA) in M9 buffer. (A) L3i feeding, a hallmark of activation, was assessed by ingestion of a FITC dye for worms incubated at 37°C and 5% CO_2_ in air for 24 hours. Error bars represent ±1 standard deviation (SD). (B-H) Transcript abundance patterns for *Ss-tgh-1* through *-7* were determined by RNAseq for each condition. Transcript abundances were calculated as fragments per kilobase of coding exon per million fragments mapped (FPKM). Error bars represent ±95% confidence intervals. All statistically significant differences, with respect to the no stimulation condition, are marked with an asterisk.
(TIF)

Data S1 ***Strongyloides stercoralis*** **homolog genome annotations.**
(TXT)

Data S2 ***Strongyloides stercoralis*** **homolog transcript sequences.**
(FA) Click here for additional data file.

Data S3 ***Strongyloides stercoralis*** **homolog predicted protein sequences.**
(FA) Click here for additional data file.

Data S4 **FPKM values for** ***Strongyloides stercoralis*** **gene coding sequences.**
(XLS)

Data S5 **Accession numbers for** ***Caenorhabditis elegans*** **protein sequences.**
(XLS)

Data S6 **Protein alignment for G protein alpha subunits.**
(TXT)

Data S7 **Primer sequences.**
(XLS)
