# Supplementary figures and images for: cGMP and NHR Signaling Co-regulate Expression of Insulin-Like Peptides and Developmental Activation of Infective Larvae in Strongyloides stercoralis
Source: PLoS Pathog. 2014 Jul 10;10(7):e1004235. doi: 10.1371/journal.ppat.1004235 (PMC4092141; doi:10.1371/journal.ppat.1004235)

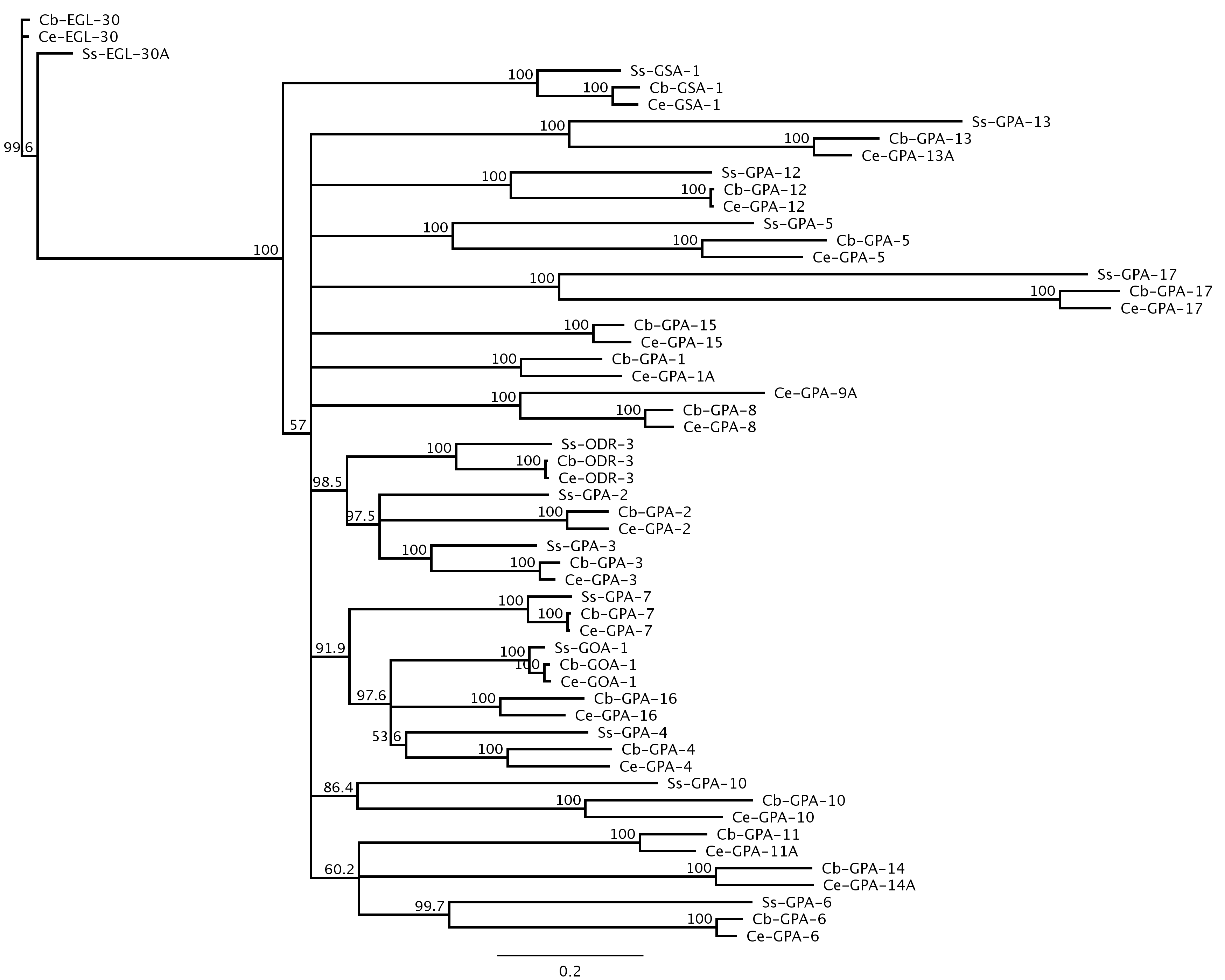

Supplement: Figure S1 — Phylogenetic analysis of S. stercoralis and Caenorhabditis spp. Gα proteins. A protein alignment, generated with Clustal W, of S. stercoralis (Ss), C. briggsae (Cb), and C. elegans (Ce) heterotrimeric G protein α subunit (Gα) homologs was used to construct a neighbor-joining tree with 100 iterations of boot-strapping. Orthologs for several C. briggsae and C. elegans Gα-encoding genes (gpa-1, -8, -9, -11, -14, -15, and -16) were not identified in the S. stercoralis draft genome or de novo assembled transcripts. The scale bar represents substitutions per position. (TIF) [file ppat.1004235.s001.tif]

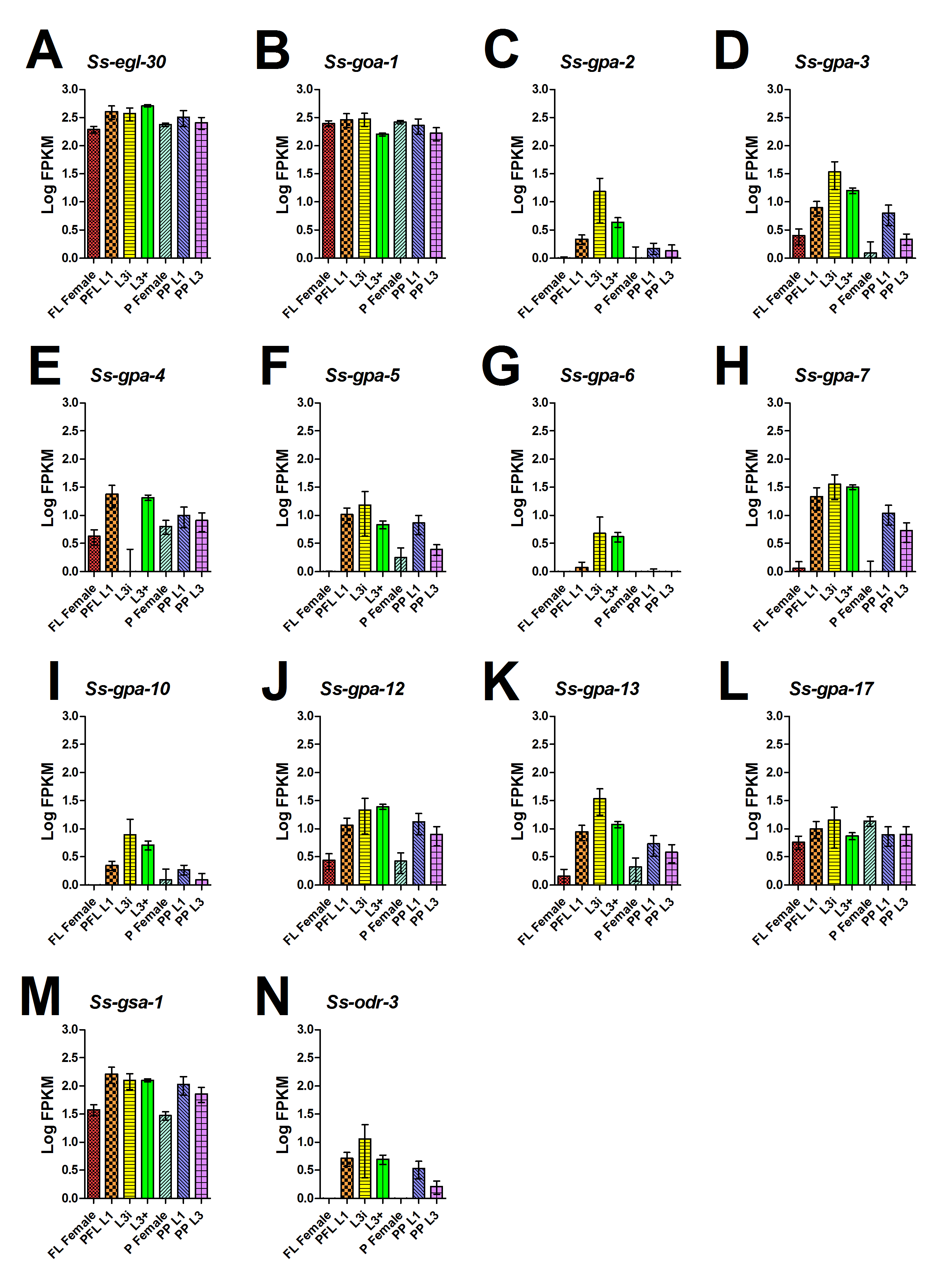

Supplement: Figure S2 — Developmental regulation of transcripts encoding S. stercoralis Gα subunits. (A–N) Transcript abundance patterns in S. stercoralis developmental stages were determined by RNAseq for genes encoding orthologs of heterotrimeric G protein α subunits (Gα). Transcript abundances were quantified in seven developmental stages: free-living females (FL Female), post-free-living first-stage larvae (PFL L1), infectious third-stage larvae (L3i), in vivo activated third-stage larvae (L3+), parasitic females (P Female), post-parasitic first-stage larvae (PP L1), and post-parasitic third-stage larvae (PP L3). Transcript abundances were calculated as fragments per kilobase of coding exon per million fragments mapped (FPKM) and log transformed. Error bars represent ±95% confidence intervals. The y-axes were scaled from 0 to 3.0 to aid comparison between genes. (TIF) [file ppat.1004235.s002.tif]

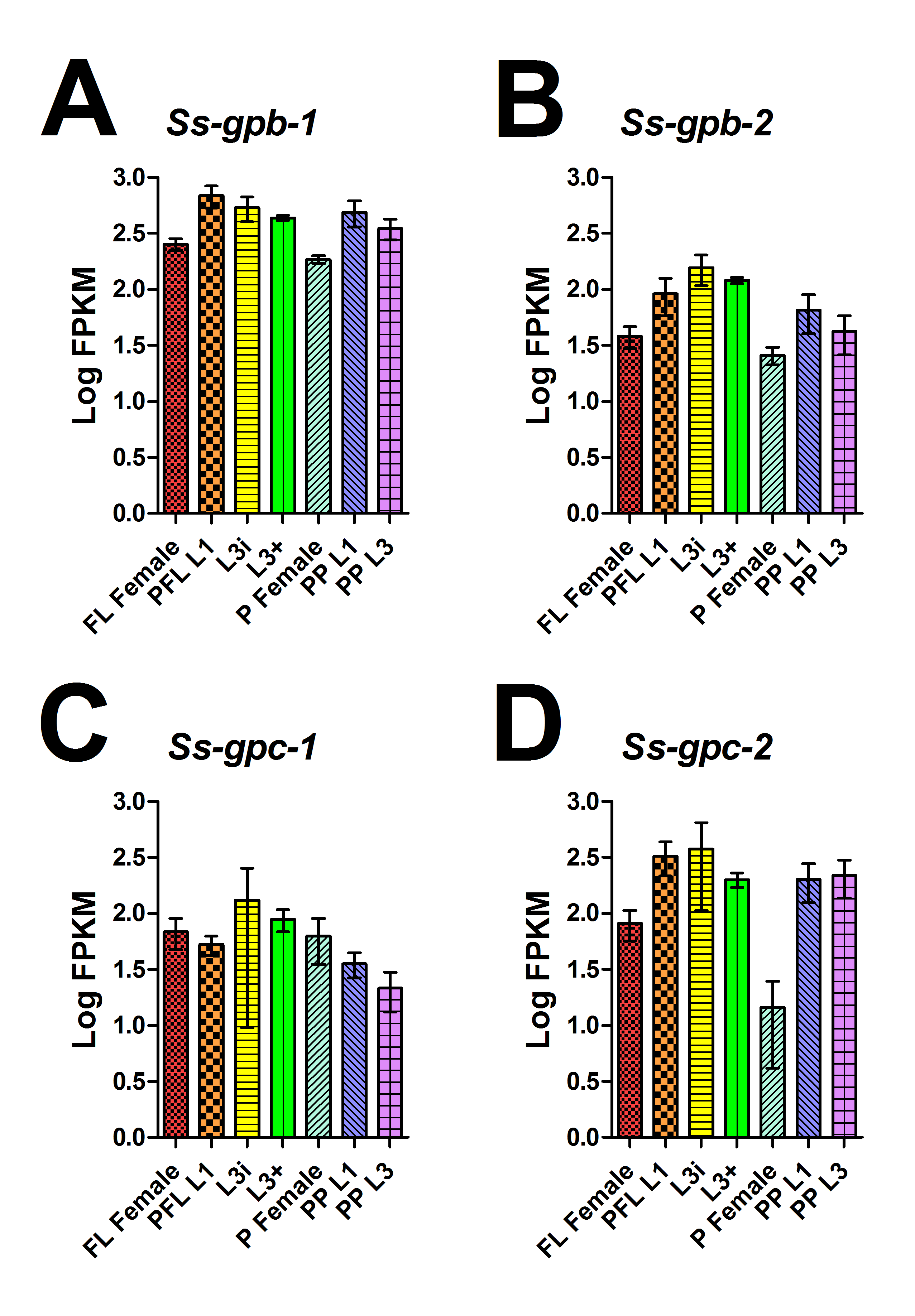

Supplement: Figure S3 — Developmental regulation of transcripts encoding S. stercoralis Gβ and Gγ subunits. (A–D) Transcript abundance patterns in S. stercoralis developmental stages were determined by RNAseq for genes encoding homologs of heterotrimeric G protein β (Gβ) subunits (A,B) and γ (Gγ) subunits (C,D). Transcript abundances were quantified in seven developmental stages: free-living females (FL Female), post-free-living first-stage larvae (PFL L1), infectious third-stage larvae (L3i), in vivo activated third-stage larvae (L3+), parasitic females (P Female), post-parasitic first-stage larvae (PP L1), and post-parasitic third-stage larvae (PP L3). Transcript abundances were calculated as fragments per kilobase of coding exon per million fragments mapped (FPKM) and log transformed. Error bars represent ±95% confidence intervals. The y-axes were scaled from 0 to 3.0 to aid comparison between genes. (TIF) [file ppat.1004235.s003.tif]

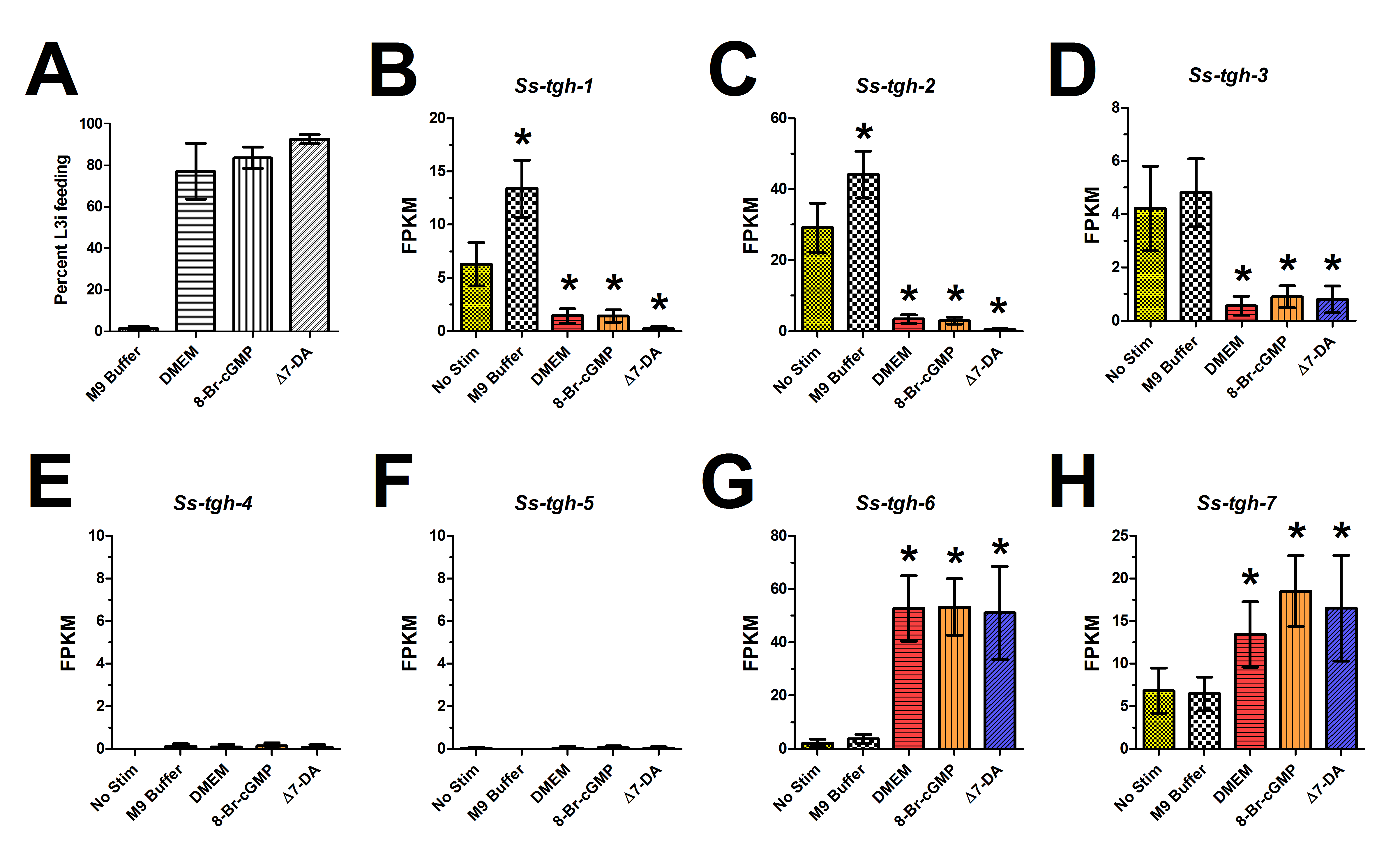

Supplement: Figure S4 — S. stercoralis L3i activation with 8-bromo-cGMP, Δ7-DA, or DMEM modulates TGFβ ligand transcript levels. Transcript levels of the DAF-7-like transforming growth factor β (TGFβ) ligand-encoding genes Ss-tgh-1 through Ss-tgh-7 were quantified using RNAseq. Conditions included L3i that had no stimulation (only exposed to room temperature conditions in M9 buffer) as well as L3i incubated at 37°C and 5% CO2 in air for 24 hours in either M9 buffer, DMEM, 200 µM 8-bromo-cGMP in M9 buffer, or 400 nM Δ7-dafachronic acid (DA) in M9 buffer. (A) L3i feeding, a hallmark of activation, was assessed by ingestion of a FITC dye for worms incubated at 37°C and 5% CO2 in air for 24 hours. Error bars represent ±1 standard deviation (SD). (B-H) Transcript abundance patterns for Ss-tgh-1 through -7 were determined by RNAseq for each condition. Transcript abundances were calculated as fragments per kilobase of coding exon per million fragments mapped (FPKM). Error bars represent ±95% confidence intervals. All statistically significant differences, with respect to the no stimulation condition, are marked with an asterisk. (TIF) [file ppat.1004235.s004.tif]
